# Supplementary material for: Preliminary Research on the Effect of Hyperbaric Oxygen Therapy in Patients with Post-COVID-19 Syndrome
Source: J Clin Med. 2022 Dec 30;12(1):308. doi: 10.3390/jcm12010308 (PMC9821575; doi:10.3390/jcm12010308)
Supplement: Supplementary file 1 [file jcm-12-00308-s001.zip › Figure S1.pdf]

Supplement 2.

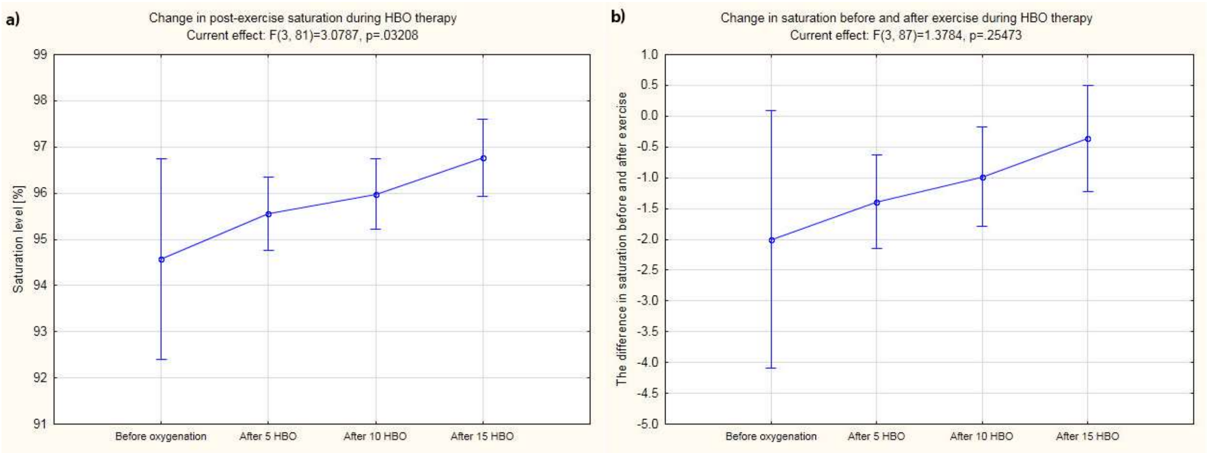

A) Results of the saturation test in patients undergoing the study: a.) Change in post-exercise saturation during HBO therapy; b.) change in the difference in saturation pre-exercise and post-exercise after 5, 10, and 15 compressions.

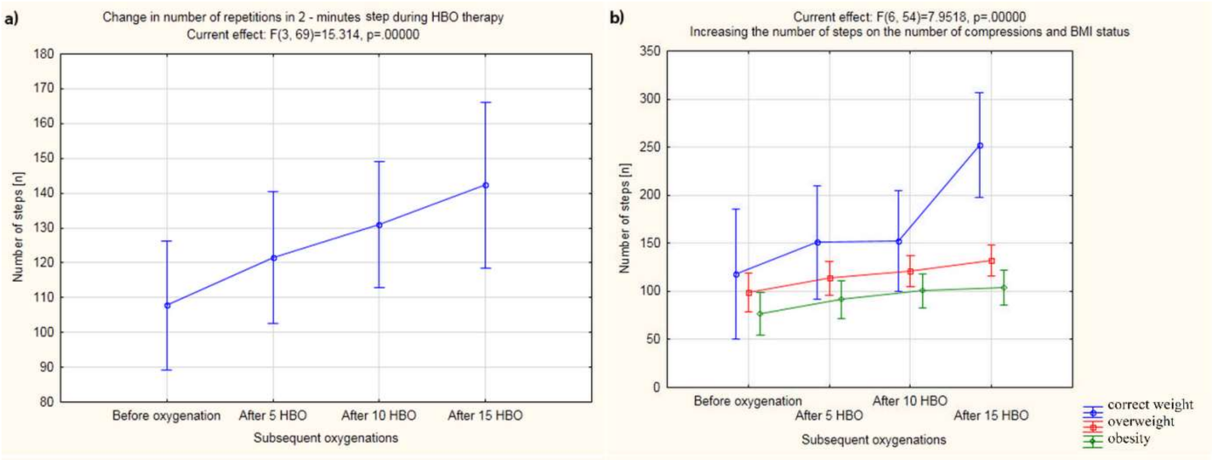

B) Two-minute step test results: a.) Change in number of steps after successive sets of compressions; b.) the effect of BMI on increasing the number of steps during the course of therapy;

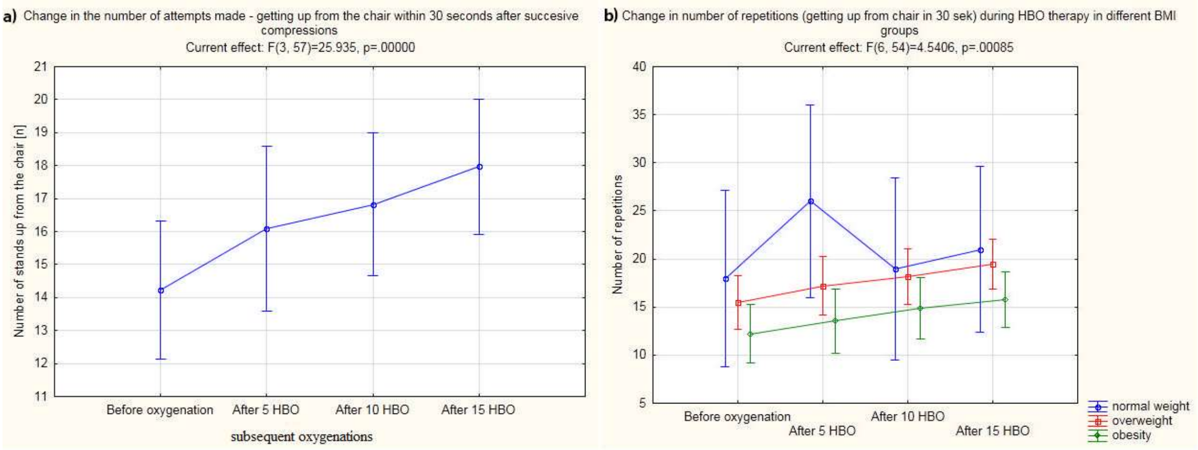

C) Chair Rising Test Results in 30 seconds: a.) Change in the number of chair lifts in 30 seconds during KBO therapy; b.) the impact of BMI on the attempt to "get up from the chair within 30 seconds".

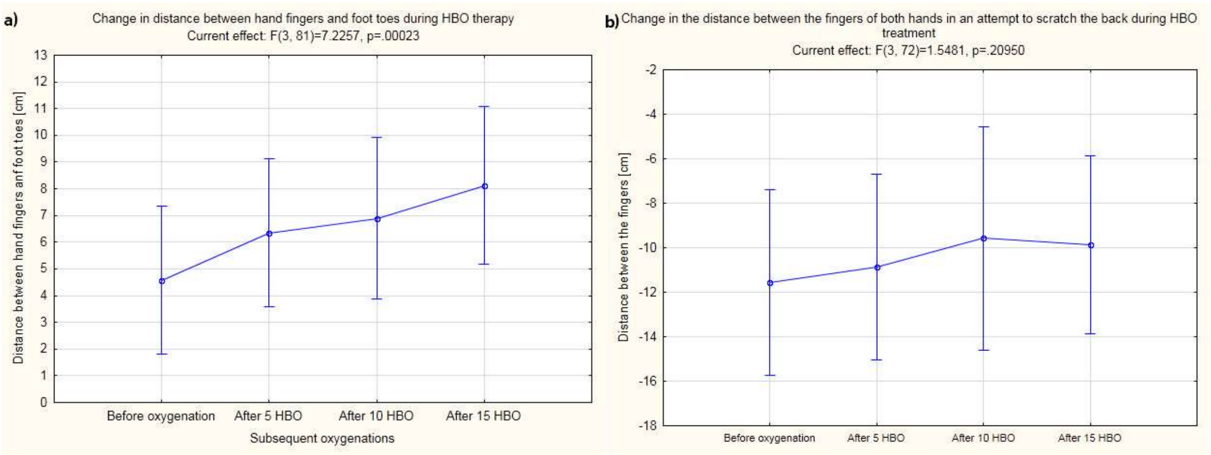

D) Sit and Reach test results - a.) the number of cm of difference between the fingers and toes in sitting down after 5, 10 and 15 compressions; b.) Non-significant change in back scratch test score with HBO treatment.

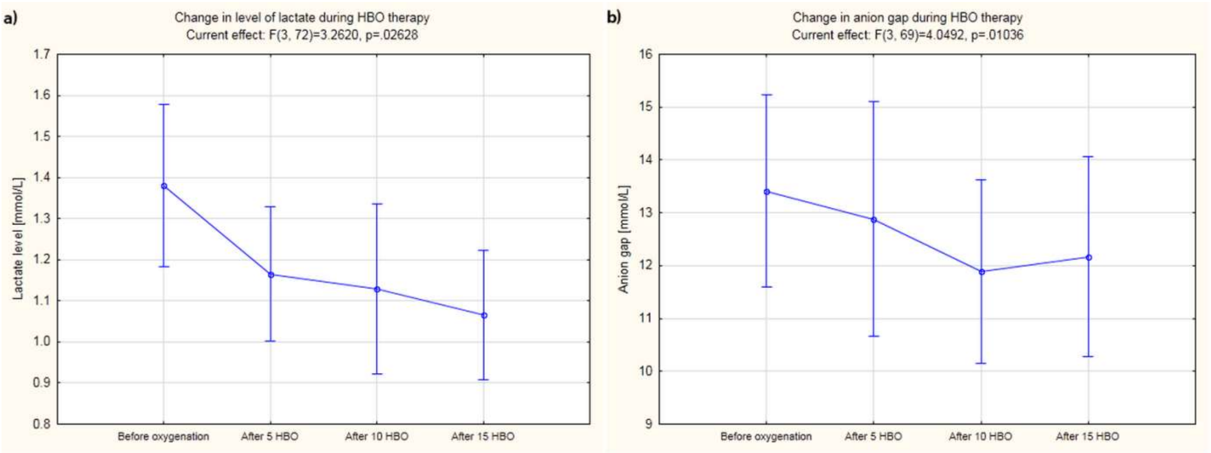

E) Results of venous blood gas tests performed in patients subjected to the test in subsequent phases of the experiment (before the start of the experiment, after 5, after 10 and after 15 compressions): a.) Lactate level; b.) anion gap.

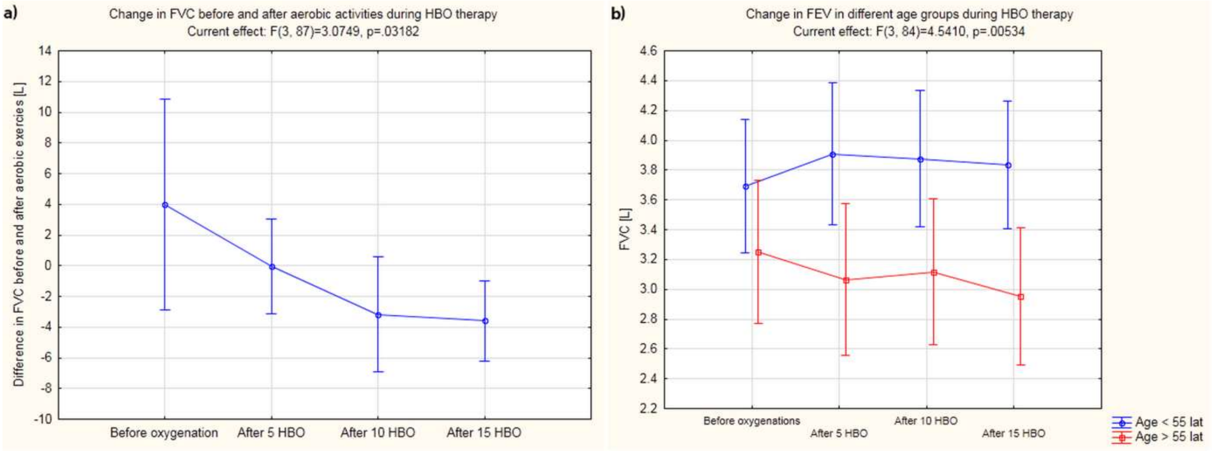

F) Results of spirometric tests in patients subjected to the study in the subsequent phases of the experiment (before the start of the experiment, after 5, after 10 and after 15 compressions): a.) FVC; b.) difference between pre-exercise and post-exercise FVC in two age groups.

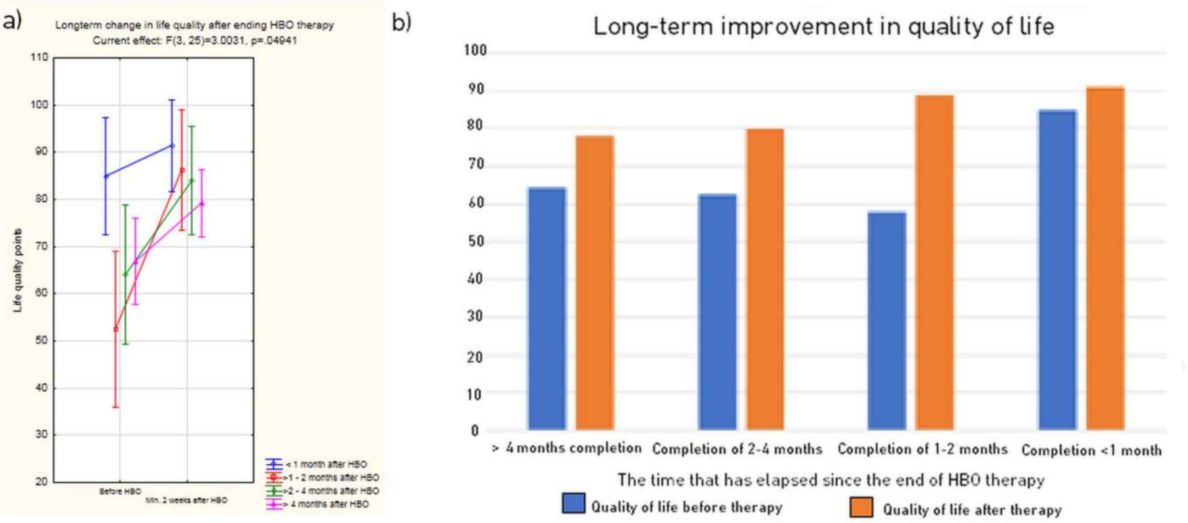

G) Results of the telephone questionnaire survey conducted on a group of patients undergoing a medical experiment after a specified period of time from the end of the study.

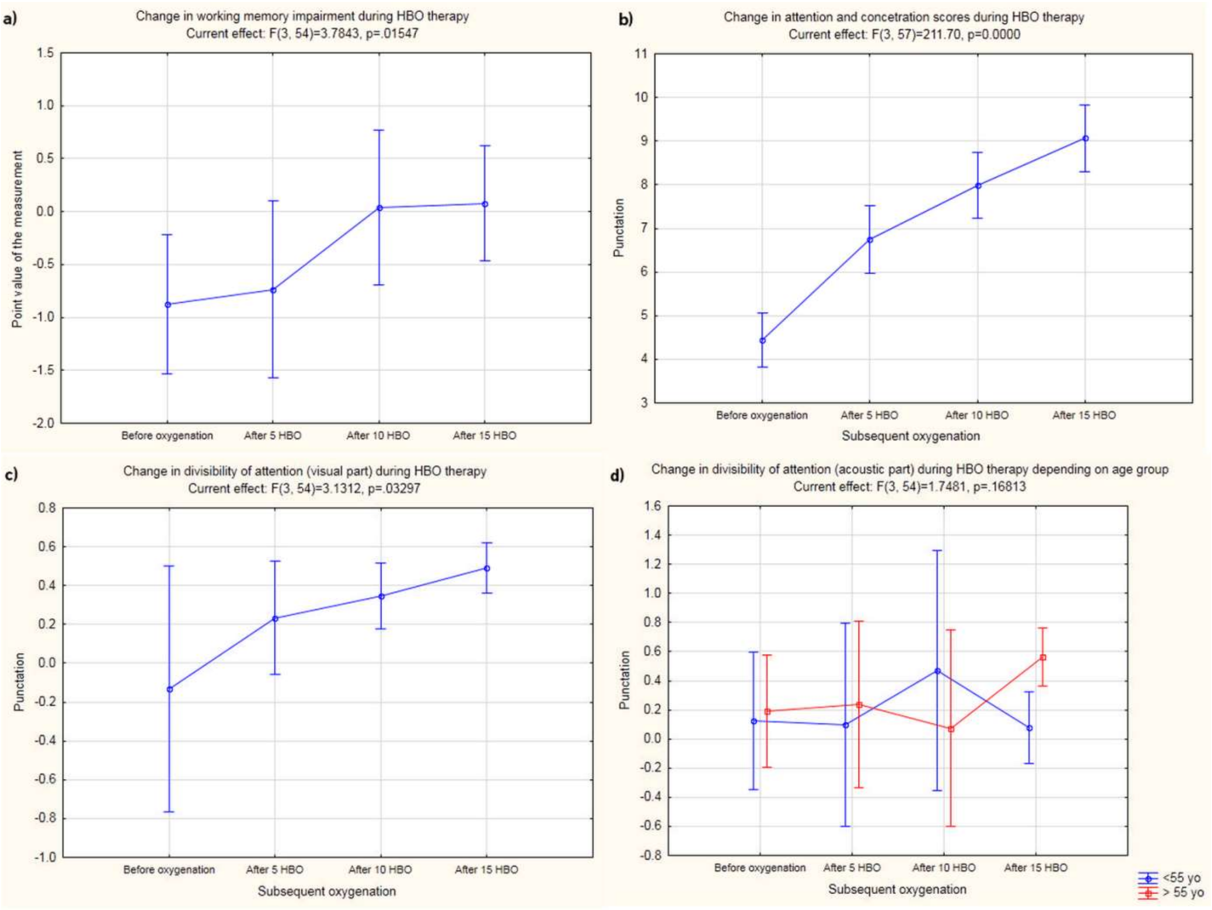

H) Results of psycho-technical tests among patients subjected to the study in subsequent phases of the experiment (before the start of the experiment, after 5, after 10 and after 15 compressions): a.) Level of working memory; b.) level of concentration and attention; c.) divided attention - visual method; d.) level of attention - acoustic method.
