# Supplementary material for: Preliminary Research on the Effect of Hyperbaric Oxygen Therapy in Patients with Post-COVID-19 Syndrome
Source: J Clin Med. 2022 Dec 30;12(1):308. doi: 10.3390/jcm12010308 (PMC9821575; doi:10.3390/jcm12010308)
Supplement: Supplementary file 1 [file jcm-12-00308-s001.zip › Table S1.pdf]

Supplement 1. The results of pulse oximetry in patients undergoing a series of HBO compressions.

	Before HBO compression		After 5 HBO sessions		After 10 HBO sessions		After 15 HBO sessions	
	**Before the ef fort/exercise**	**After the ef fort/exercise**	**Before the ef fort/exercise**	**After the ef fort/exercise**	**Before the ef fort/exercise**	**After the ef fort/exercise**	**Before the ef fort/exercise**	**After the ef fort/exercise**
Saturation:<br>- Mean<br>- Minimal	96.8 ± 1.9<br>93	94.8 ± 5.4<br>70	97.1 ± 1.9<br>91	95.7 ± 2.2<br>90	97.1 ± 1.7<br>92	96.2 ± 2.2<br>89	97.2 ± 1.7<br>92	96.8 ± 2.2<br>90
Mean difference between post-exercise and pre-exercise oxygen saturation	-2.03		-1.39		-0.97		-0.35	
Pulse:<br>- Average<br>- Min.<br>- Max.<br>- mode	75.3 ± 12<br>55<br>100<br>76	92.8 ± 18.4<br>31<br>124<br>90	71.4 ± 10.4<br>52<br>88<br>87	94 ± 16.7<br>65<br>123<br>104	71.3 ± 10<br>54<br>89<br>74	90.9 ± 17.2<br>64<br>126<br>86	70.2 ± 11.2<br>50<br>96<br>75	92.3 ± 16.9<br>61<br>122<br>105
Average difference between post-exercise and pre-exercise heart rate	17.5 ± 6.4		22.6 ± 6.3		19.7 ± 7.2		22.1 ± 5.7	
