# Supplementary material for: Preliminary Research on the Effect of Hyperbaric Oxygen Therapy in Patients with Post-COVID-19 Syndrome
Source: J Clin Med. 2022 Dec 30;12(1):308. doi: 10.3390/jcm12010308 (PMC9821575; doi:10.3390/jcm12010308)
Supplement: Supplementary file 1 [file jcm-12-00308-s001.zip › Table S2.pdf]

Supplement 3. Fullerton test results in patients tested during the HBO therapy.

	Before HBO compression	After 5 HBO sessions	After 10 HBO sessions	After 15 HBO sessions
Getting up from a chair in 30 seconds:				
- Mean	13.9 ± 4.3	16.1 ± 5.1	17.3 ± 5	18.5 ± 4.9
- Min.	5	6	7	9
- Max.	25	28	31	33
- Mode	11	16	19	19
8 Foot Test - Stand Up and Walk (seconds):				
- Mean	6.7 ± 2.5	6.1 ± 2.8	5.9 ± 2.5	5.5 ± 1.9
- Min.	4.4	3.9	3.8	3.8
- Max.	16	18	16.5	13.3
- Mode	4.9	5.8	4.3	3.8
Two minute step test				
- Mean	92.2 ± 31.8	107.5 ± 31.3	113.7 ± 31.6	126 ± 38.7
- Min.	32	40	52	56
- Max.	150	163	177	252
- Mode	68	103	75	91
Dynamometric test (N):				
- Left hand:				
- Mean	28.4 ± 11.9	29.9 ± 12.6	30.1 ± 13	32.7 ± 13.5
- Min.	3.75	4	4	4
- Max.	49	51	52	68
- Mode	21	32	44	24
- Right hand:				
- Mean	29.4 ± 11.5	30.1 ± 12.1	32.3 ± 12.1	33.3 ± 12.9
- Min.	6.25	4	2	4
- Max.	55	53	54	65
- Mode	22	22	38	36
Back scratch test (cm):				
- Mean	−9.6 ± 12.9	−9.4 ± 11.7	−8.5 ± 11.7	−8.6 ± 9.7
- Min.	−29	−26	−25.50	−25.5
- Max.	37.5	26	30	7
- Mode	−7	−24	−11.5	−10
Try to sit down and reach (cm):				
- Mean	4 ± 7.2	6 ± 7.3	6.1 ± 8.1	8 ± 7.6
- Min.	−10	−7	−11	−7
- Max.	21	20	21	20
- Mode	0	3	0	0
