# Supplementary material for: Preliminary Research on the Effect of Hyperbaric Oxygen Therapy in Patients with Post-COVID-19 Syndrome
Source: J Clin Med. 2022 Dec 30;12(1):308. doi: 10.3390/jcm12010308 (PMC9821575; doi:10.3390/jcm12010308)
Supplement: Supplementary file 1 [file jcm-12-00308-s001.zip › Table S3.pdf]

Supplement 4. The results of venous blood gasometry in patients subjected to the test in the subsequent phases of the experiment (before the start of the experiment, after 5, after 10 and after 15 compressions).

	**Before HBO compression**	**After 5 HBO sessions**	**After 10 HBO sessions**	**After 15 HBO sessions**
pCO_2				
- Mean	47.8 ± 5.1	51.2 ± 5.7	50.7 ± 7.2	66.6 ± 8.7
- Min.	37	42.4	38.2	22.5
- Max.	56.6	62.3	66.2	68.8
pO_2				
- Mean	31 ± 10	25.65 ± 7.2	28.05 ± 11.6	28.6 ± 8.6
- Min.	20.2	14.6	18	18.6
- Max.	62.6	44.8	73.8	60.3
cBASE				
- Mean	−3.34 ± 5.4	−2.74 ± 5.6	−2.6 ± 5.8	−3.43 ± 5.3
- Min.	−11	−10	−12	−16.6
- Max	5.1	7.7	7.8	5.4
pH				
- Mean	7.28 ± 0.1	7.27 ± 0.07	7.28 ± 0.07	7.27 ± 0.05
- Min.	7.14	7.15	7.13	7.16
- Max	7.4	7.42	7.44	7.36
Anion gap:				
- Mean	13.5 ± 4.2	12.7 ± 4.8	11.7 ± 4.2	12.6 ± 4.4
- Min.	6.4	5.2	5.2	2.7
- Max	18.7	23.4	17.9	18.8
Lactates				
- Mean	1.35 ± 0.47	1.15 ± 0.41	1.11 ± 0.48	1.06 ± 0.4
- Min.	0.6	0.3	0.4	0.5
- Max	2.5	2.3	2.3	1.8
Glucose:				
- Mean	124 ± 42	117.8 ± 28.7	115.7 ± 30.5	112 ± 8.8
- Min.	90	90	82	87
- Max	287	215	242	169
